# Tocotrienols: Dietary Supplements for Chronic Obstructive Pulmonary Disease

**DOI:** 10.3390/antiox10060883

**Published:** 2021-05-31

**Authors:** Xiangming Ji, Hongwei Yao, Maureen Meister, Douglas S. Gardenhire, Huanbiao Mo

**Affiliations:** 1 Department of Nutrition, Byrdine F. Lewis College of Nursing and Health Professions, Georgia State University, Atlanta, GA 30303, USA; mbeebe@student.gsu.edu (M.M.); hmo@gsu.edu (H.M.); 2 Department of Molecular Biology, Cell Biology & Biochemistry, Division of Biology and Medicine, Brown University, Providence, RI 02912, USA; hongwei_yao@brown.edu; 3 Department of Respiratory Therapy, Byrdine F. Lewis College of Nursing and Health Professions, Georgia State University, Atlanta, GA 30303, USA; dgardenhire@gsu.edu

**Keywords:** COPD, tocotrienols, antioxidant, anti-inflammation

## Abstract

Chronic obstructive pulmonary disease (COPD) is one of the leading causes of death worldwide. Emphysema and chronic bronchitis are the two major phenotypes of COPD, which have many symptoms, such as dyspnea, chronic cough, and mucus overproduction. Emphysema is characterized by the destruction of the alveolar wall, while chronic bronchitis is characterized by limitations in expiratory airflow. Cigarette smoking is the most significant risk factor for the pathogenesis of COPD in the developed world. Chronic inflammation contributes to the onset and progression of the disease and furthers the risk of comorbidities. Current treatment options and prevention strategies for COPD are very limited. Tocotrienols are a group of vitamin E molecules with antioxidant and anti-inflammatory properties. Individual tocotrienols (α, γ, and δ) have shown their ability to attenuate inflammation specifically via suppressing nuclear factor-κB-mediated cytokine production. The δ- and γ-forms of tocotrienols have been indicated as the most effective in the prevention of macrophage infiltration, production of reactive oxygen species, and cytokine secretion. This review briefly discusses the pathogenesis of COPD and the role of inflammation therein. Furthermore, we summarize the in vitro and in vivo evidence for the anti-inflammatory activity of tocotrienols and their potential application to COPD management. Coupled with the bioavailability and safety profile of tocotrienols, the ability of these compounds to modulate COPD progression by targeting the inflammation pathways renders them potential candidates for novel therapeutic approaches in the treatment of COPD patients.

## 1. Introduction

Chronic obstructive pulmonary disease (COPD) is currently listed as the 4th leading cause of death, with 328 million patients diagnosed worldwide [1]. COPD is characterized by limitations in expiratory airflow, the emphysematous destruction of the lungs, chronic bronchitis, and the chronic inflammation of lung tissue. Symptoms of COPD include dyspnea, productive cough, and mucus overproduction. Comorbid conditions such as cardiovascular disease and lung cancer reduce the survival rate of COPD patients [2]. Cigarette smoking is a major risk factor for the pathogenesis of COPD in developed countries.

Although the prevalence of COPD continues to increase, strategies for the prevention, treatment, and management of the disease are limited. Current treatment options for COPD include bronchodilators, such as long-acting ß_2_-adrenergic receptor agonists (LABA) and long-acting muscarinic receptor antagonists (LAMA). These medications need to be taken daily for the prevention of disease exacerbations, a condition in which COPD increases in severity for a period of time [3]. However, neither LABA nor LAMA can reduce the oxidative stress and inflammatory state of the disease without severe side effects [4]. It is recommended that corticosteroid inhalers should be given only if bronchodilator therapy fails to control symptoms [5]. Although systemic and inhaled corticosteroids assist in the prevention and treatment of COPD exacerbations, they provide little therapeutic benefit in patients with stable COPD [6,7]. In addition, corticosteroids cause multiple side effects in patients with COPD, such as higher risks of developing pneumonia and osteoporosis, easy bruising, and thinning of the skin [2,8]. The dearth of weaponry in the arsenal to tackle COPD calls for new options.

This review provides new insights on the pathogenesis, diagnosis, risk factors, and molecular mechanisms of COPD with an emphasis on the role of oxidative stress and inflammation. Because of the persistent role of oxidative stress and inflammation in the development of the disease, we postulate that antioxidant and anti-inflammatory agents, notably the vitamin E molecules as evidenced by in vitro, in vivo, and human studies, have potential in COPD prevention.

## 2. Pathogenesis, Diagnosis, and Risk Factors of COPD

Chronic bronchitis and emphysema are the two most common clinical conditions of COPD. Chronic bronchitis is caused by the existence of inflammation within airway bronchial tubes, which leads to the narrowing of the small airways that carry air in and out of the air sacs (alveoli) in the lungs. Patients with chronic bronchitis often experience mucus accumulation in the lung and chronic cough [9]. The mucus is mainly produced by goblet cells, which interact with the immune system and serve as the first line of defense for the respiratory tract. Chronic inflammation causes epithelial injuries and the loss of lung elasticity, which makes it difficult to clear the mucus from the airway. Patients with COPD have an increased number of goblet cells and inflammatory cells within the airway epithelium, contributing to mucus hypersecretion [10]. Besides, damaged cilia in the epithelial lining also hinder the clearance of the mucus in the lung. Therefore, the accumulation of mucus and an unproductive cough due to respiratory muscle weakness exacerbate symptoms of COPD. Emphysema, also known as parenchymal destruction, is characterized by the abnormal, permanent enlargement of air spaces distal to the terminal bronchiole, resulting in a loss of alveolar attachment and a decrease in the elasticity of the lungs [2]. These changes keep the lungs from remaining open during expiration. Limited airflow and reduced expiratory output result in dyspnea or labored breathing, a common symptom of COPD.

Spirometry is a common pulmonary function test to diagnose and identify the stage of COPD. Two main criteria are measured by spirometry: the forced expiratory volume in one second (FEV_1_), which is the amount of air an individual is able to force from the lungs in one second; and the forced vital capacity (FVC), which is the greatest volume of air that can be breathed out in a single large breath [11]. The ratio of FEV_1_/FVC, also called the Tiffeneau-Pinelli index, is regularly utilized in the diagnosis of COPD [12]. As approximately 80% of the FVC is expelled within the first second, a ratio of FEV_1_/FVC less than 70% is considered an indicator of COPD [13]. The Global Initiative for Chronic Obstructive Lung Disease (GOLD) guidelines divide COPD patients into four categories based on symptom assessment and airflow limitation evaluation [14].

There are many risk factors for COPD initiation, progression, and exacerbation. These risk factors include genetic predisposition, impaired lung development, early-life lung injury, and exposure to occupational hazards and environmental toxins. While genetic risk factors are responsible for about 2% of COPD cases [15], cigarette smoking is the most important and the most researched risk factor for the initiation and development of COPD [2]. About 50% of COPD patients have smoking history [16,17]. Moreover, a longitudinal study confirmed that 32% of ever-smokers exhibited a decline in FEV_1,_ indicating an overall impaired lung function [18]. In addition, other types of tobacco exposure, such as electronic-cigarette smoking/vaping, marijuana use, and passive exposure to cigarette smoke serve as risk factors for COPD as well [2]. Previous studies have shown that, compared with individuals who never smoked, smokers and ex-smokers with symptoms of chronic bronchitis had a higher mortality risk (hazard ratio 2.89 and 1.69, respectively) [19].

## 3. Oxidative Stress in the Lung

Due to its unique structure, the lung is very sensitive to oxidative stress by the environment. As aforementioned, free radicals from cigarette smoking lead to an oxidative microenvironment in the lungs. Chronic exposure to this reactive microenvironment will cause epithelial damage due to the presence of overwhelming reactive oxygen species (ROS) [20]. During acute exacerbations, the total number of alveolar macrophages is elevated, which produces superoxide radicals, hydrogen peroxides, and other forms of ROS in patients with COPD [21]. Similarly, activated neutrophils also release increased amounts of ROS during exacerbations. The aggregation of all these ROS can activate the nuclear factor-κB (NFκB) pathway. The activation of the NFκB pathway and the production of cytokines correlate with airflow limitation in patients [22].

## 4. Biological Mechanisms of COPD: Free Radicals, Inflammation, and the NFκB Pathway

**Figure 1:**
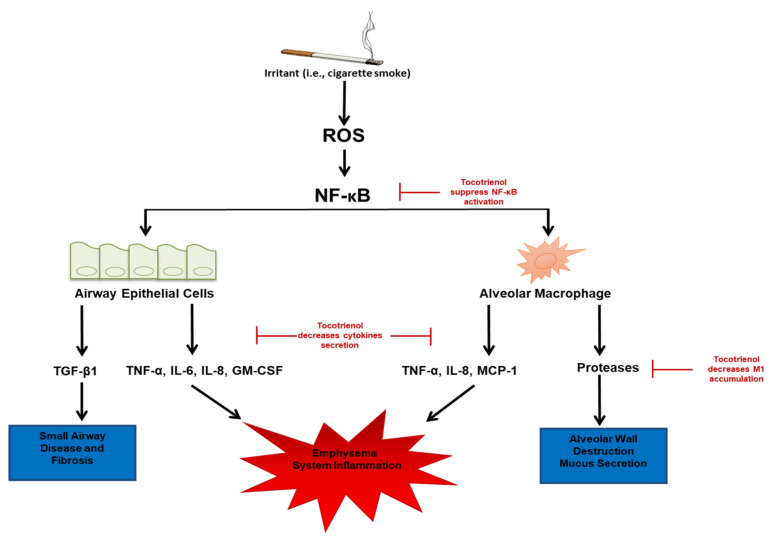
The effects of tocotrienols on the pathogenesis of COPD. Environmental irritants, such as cigarette smoke, produce ROS that activates NFκB, a major inflammation regulator. NFκB, in turn, stimulates the production of pro-inflammatory cytokines including TGF-β1, TNF-α, IL-6, IL-8, and GM-CSF in airway epithelial cells and TNF-α, IL-8, MCP-1, and proteases in alveolar macrophages. Collectively, these pro-inflammatory molecules lead to small airway disease and fibrosis, alveolar wall destruction, mucus secretion, emphysema, and systemic inflammation. Tocotrienols suppress NFκB activation, decrease cytokine secretion and M1 accumulation, and consequently attenuate the inflammatory response induced by the irritants.

Cigarette smoke contains more than 7000 chemical compounds, among which 69 carcinogens have been identified [23]. As a major risk factor for COPD, cigarette smoke is a significant source of two major types of free radicals: tar and gas-phase free radicals [24,25]. Inhalation of these free radicals leads to lung injury either by direct tissue oxidation or by the indirect recruitment and activation of neutrophils and macrophages [26,27]. Furthermore, exposure to these free radicals also amplifies the effects of neutrophil protease, resulting in the destruction and degradation of the alveolar lung in smokers [28]. As shown in Figure 1, exposure to these free radicals leads to epithelial damage and a decrease in airway epithelial barrier function [29]. This change makes the patients vulnerable to bacterial and viral infections in COPD, both by facilitating the access of pathogens as well as by impairing the recovery of the mucosal immune barrier upon pathogen-induced damage. Bearing the harmful compounds, cigarette smoking causes acute and chronic inflammatory responses, which make individuals susceptible to the development of COPD [30]. It is not surprising that these free radicals within cigarette smoke exacerbate disease development and progression [31]. Moreover, a higher level of oxidative stress lingers in the respiratory system even after smoking cessation [20]. In accordance, the serum level of the antioxidant vitamin C is inversely associated with the smoking-related risk of COPD [32]. Therefore, targeting oxidative stress pathways with antioxidants would be beneficial in patients with COPD.

As shown in Figure 1, cigarette smoke consists of oxidants that contribute to the surplus of reactive oxygen species (ROS) present in lung tissue, exacerbating the activation of the NFκB pathway, a key regulator of inflammation, during the development of COPD. ROS from cigarette smoke can activate NFκB through novel endogenous ligands, such as toll-like receptors (TLRs), providing a key link between oxidative stress and innate immunity [33]. Cigarette smoking also increases the expression of TLR4 and lipopolysaccharide (LPS) binding, further promoting NFκB activation [34]. The inhalation of noxious particles from other environmental and occupational sources aggravates lung inflammation. This chronic inflammatory response leads to the destruction of parenchymal tissue and the stiffening of small airways, resulting in the decreased elasticity of the lung tissue for expiration, which is characterized as emphysema.

In addition to free radicals, localized inflammation within the lung also plays a role in COPD progression [35]. The inflammatory response is a defense mechanism that aims to remove the initial cause of cell injury, clear out damaged cells and tissues, and initiate tissue repair. Inflammation can be classified as either acute or chronic inflammation. Acute inflammation is a short-term process (from minutes to hours) and begins to cease upon the removal of the injurious stimulus [36], whereas chronic inflammation is a slow process lasting for prolonged periods of several months to years. The continuous presence of irritants in the respiratory tract such as cigarette smoke or pollutants induces chronic inflammation at mucosal surfaces and finally modifies host responses to exogenous antigens [37]. These inhaled irritants activate and direct the macrophages in the pulmonary alveolus surface to the location of injury to engulf and react to the irritant/pollutant [38].

In addition, as shown in Figure 1, the activated inflammation recruits macrophages and neutrophils, as well as T and B lymphocytes. Combined with ROS, neutrophils and activated macrophages present within the lung tissue release additional cytokines and proteases, which cause inflammation and the destruction of alveoli. Moreover, the presence of irritants and the progression of COPD also contribute to the activation of M1 and M2 macrophages [38], leading to macrophage polarization. Both biomarkers of M1 and M2 macrophages are elevated in the tissues of COPD patients, as well as in the sputum of smokers [39]. All these changes propel the status of inflammation, allowing for the progression of the disease and increased risks of comorbidities.

Chronic inflammation in COPD is associated with profound metabolic reprogramming, particularly through the activation of the NFκB pathway [40]. Using metabolic flux analyses of [U-^13^C_5_] glutamine, our recent study demonstrated that long-term exposure to cigarette smoke condensate (CSC) increased lipid biosynthetic capacity and glutamine reductive carboxylation [40]. This metabolic reprogramming is highly associated with interleukin-6 (IL-6) and IL-8 production (unpublished data), which is regulated by the NFκB pathway. This observation is consistent with previous findings that free radical-containing cigarette smoke promotes the pro-inflammatory NFκB pathway [41]. Additionally, the neutrophils in the sputum from the patients with COPD show increased NF-κB signaling following exposure to cigarette smoke (CS) extract [42].

NFκB plays a key role in regulating the accumulation of pro-inflammatory cytokines in the respiratory tract and mucus during the development of COPD. Circulating cytokines, including C-reactive protein (CRP), tumor necrosis factor α (TNF-α), IL-6, and IL-8, have been considered biomarkers of COPD [43]. IL-6 activates CRP, which is also associated with the progression and severity of COPD [44]. IL-8 is found in significantly high amounts in the mucosa of patients with COPD [45,46]. As the key chemokine produced by macrophages, IL-8 attracts neutrophils, B cells, and T cells to the site, further promoting the inflammatory response. Furthermore, TNF-α, a cytokine that activates the NFκB-mediated pro-inflammatory pathway, is overproduced in the sputum of COPD patients and even higher amounts during an episode of COPD exacerbation. Cigarette smoking increases the productions of cytokines such as IL-1β, IL-6, IL-8, IL-17, and granulocyte-colony stimulating factor (G-CSF) [44]. These cytokines are produced by the airway epithelial cells and the macrophages activated within lung tissue (Figure 1). Under the regulation of the NFκB pathway [45,46], epithelial cells and alveolar macrophages also induce neutrophil infiltration in the lungs. The presence of neutrophils amplifies the level of inflammation [47]. Besides, many other inflammatory markers are also found in circulation, forming systemic inflammation in lung tissue. It is unknown whether this is due to a “spill-over effect” from the pulmonary tissue or if it develops in parallel with the disease itself. Altogether, the coexistence of free radicals, parenchymal inflammation, mucus hypersecretion, the activation of the NFκB pathway, and cytokine overproduction are common in most COPD patients. Therefore, identifying new compounds targeting these oxidative and inflammatory pathways simultaneously with low toxicity is crucial in developing new strategies for COPD intervention.

## 5. Role of Vitamin E in COPD: Introduction to Tocopherols and Tocotrienols

COPD patients have higher systemic and airway oxidative markers and lower plasma antioxidant levels and activity than healthy subjects [48]. This higher oxidant-to-antioxidant ratio is at least in part attributed to the activation of NFκB and inflammation [49]. Lung function was found to be positively correlated with antioxidant enzymes, including catalase, superoxide dismutase, glutathione peroxidase [50,51], antioxidant vitamins [52,53,54], and the intake of antioxidant-rich fruits and vegetables [55] but inversely correlated with oxidative stress [49,50,55].

Antioxidants are the front-line defenders against free radicals within the cells by serving as the free-radical scavengers. Vitamin E is one class of antioxidants protecting cell membranes from radical-induced injury in humans by blocking lipid peroxidation initiation and progression [56]. Vitamin E consists of two groups: tocopherols and tocotrienols, each with four distinct isomers (α, β, γ, and δ). The major difference between these two groups lies in the unsaturated hydrophobic tridecyl side chain of tocotrienols that is absent in tocopherols. The four tocotrienols differ in the number and location of the methyl group on the chromanol ring [57].

Tocopherols and tocotrienols both exhibit strong antioxidant effects. The most bioavailable vitamin E isomer in human tissues, α-tocopherol, was previously considered the most potent antioxidant vitamin E [58,59]. Yet, the role of α-tocopherol in modulating the risk of chronic disease is controversial [60,61,62,63]. Long-term supplementation with α-tocopherol failed to reduce COPD symptoms in a large cohort of male smokers randomized into the ATBC Study [64]. In comparison, recent studies have shown that tocotrienols are superior to tocopherols in their antioxidant and anti-inflammatory activities [57,65,66,67,68,69,70]. One study demonstrated that α-tocotrienol has 40~60 times higher antioxidant activity than α-tocopherol in preventing oxidative stress in rat liver microsomal membranes [66]. Tocotrienols are more evenly distributed in the phospholipid bilayer and more efficiently recycled, allowing for more effective interaction with free radicals. This merit of tocotrienols is also attributed to their preferential cellular uptake [68] and more effective penetration into fatty tissues, where they can exhibit their preventive effects [57,71].

## 6. Antioxidant and Anti-Inflammatory Effects of Tocotrienol: Cell Culture Models

α, γ, and δ-Tocotrienols are found to be more abundant among all the tocotrienols in nature and have been investigated for their role in the prevention and treatment of chronic disease [57,71]. More extensive research has been focused on δ- and γ-tocotrienols with higher anti-inflammatory activities than α-tocotrienol, as shown in Table 1.

α-Tocotrienol was one of the first tocotrienols to be investigated, as it contains the same aromatic chromanol “head” as α-tocopherol [66]. It was found that α-tocotrienol suppressed lipopolysaccharide (LPS)-induced cell death and the production of TNF-α, IL-6, and IL-8 in human A549 lung carcinoma cells [72]. Additionally, γ-tocotrienol obliterated the cigarette smoking condensate (CSC)- and TNF-α-mediated activation of the NFκB molecular pathway in KBM-5 cells, lung adenocarcinoma H1299 cells, embryonic kidney A293 cells, breast cancer MCF-7 cells, multiple myeloma U266 cells, and squamous cell carcinoma SCC4 cells [73]. On the contrary, tocopherol at a similar dose does not have the same anti-inflammatory effects [73]. These observations are consistent with the finding that dietary tocopherol did not lower COPD mortality [74].

δ-Tocotrienol, a tocotrienol isoform found in palm oil, rice bran, and annatto seeds, has shown significant antioxidant and antitumor activity [75,76]. Besides cancer cell lines, δ-tocotrienol inhibits the expression of pro-inflammatory markers such as IL-6 and NFκB in human umbilical vein endothelial cells (HUVECs) [77] and TNF-α, IL-6, and iNOS in macrophages [78]. In macrophages, annatto-derived δ-tocotrienol significantly dampens ROS production, reduces IL-1β secretion, and the NOD-like receptor family pyrin domain-containing 3 (NLRP3) inflammasome [79]. In murine RAW 264.7 macrophages and primary bone-marrow-derived macrophages, γ-tocotrienol prevented the cytokine-induced activation of NFκB by upregulating the two inhibitors of NFκB, zinc finger protein A20 and Cezanne, as well as the de novo synthesis of the sphingolipid pathway [80]. A recent study from the same group found that tocotrienol induces distinct modification of the macrophage lipidome by lowing the arachidonic acid release in macrophages from the mice [81]. The abundance of IκBα drives the degradation of NFκB, which in turn decreases the expression of pro-inflammatory markers such as CD11b, TNF-α, IL-6, and IL-1β in LPS-stimulated bone-marrow-derived macrophages and the polarization of M1 macrophages in IFNγ-treated bone marrow hematopoietic cells [82]. Molecular mechanism analysis indicates the tocotrienol-mediated inhibition of IκBα kinase, leading to the binding of IκBα (nuclear factor of kappa light polypeptide gene enhancer in B-cells inhibitor, α) to cytoplasmic p65 and eventually, the suppression of NFκB activation [73]. Cumulating evidence suggests δ-tocotrienol may also ameliorate chronic inflammation by suppressing NFκB activation [83,84]. Our unpublished data show that δ-tocotrienol decreases NFκB-DNA binding activity. γ-Tocotrienol, abundant in palm fruit, is a major component of the tocotrienol-rich factor (TRF) that is often used for inflammatory intervention studies. γ-Tocotrienol possesses a poly unsaturated tail to trap electrophiles, such as reactive nitrogen species (RNS) that are upregulated in an inflammatory state [58]. Moreover, γ-tocotrienol prevents inflammation by degrading the IκBα kinase in fully differentiated adipocytes stimulated with LPS. Additionally, δ-tocotrienol inhibits the expression of TNF and MMP-9, downstream of NFκB signaling pathways [76]. Likewise, δ-tocotrienol exerts its anti-inflammation effects by inhibiting TNFα-induced phosphorylation of transforming growth factor activated kinase 1 (TAK1) and upregulating A20 and cylindromatosis (CYLD), two inhibitors of NFκB in murine RAW 264.7 macrophages [85].

Similarly, δ-tocotrienol mediate NFκB downstream targets in osteoblastic and 3T3-L1 adipocytes cells [86,87]. δ-Tocotrienol diminishes the secretion of monocyte chemoattractant protein (MCP)-1 and IL-6 and suppresses the activation of NFκB in murine 3T3-L1 adipocytes treated with TNF-α [88]. Grapeseed oil containing significant amounts of α-tocotrienol reduces the LPS-induced production of inflammatory cytokines, including IL-6, IL-8, and monocyte chemoattractant protein 1 (MCP-1), in humans adipose-derived stem cells [89]. These findings suggest that the intake of tocotrienols by individuals with chronic diseases will be beneficial due to its potent anti-inflammatory effects.

## 7. Antioxidant and Anti-Inflammatory Effects of Tocotrienol: Animal Models

In addition to the in vitro evidence for the benefits of tocotrienols, a significant amount of research has focused on the role of vitamin E isoforms in vivo. A tocotrienol mixture suppressed the activation of antioxidant enzymes, such as glutathione reductase (GR) and glutathione peroxidase (GPx) in rats [90]. δ-Tocotrienol is more effective than other tocotrienol isoforms in reducing the serum levels of TNF-α in LPS-induced inflammation in mice, demonstrating a systemic impact of this isoform [78]. In C57BL/6J male mice fed a high-fat diet, δ-tocotrienol supplementation for 14 weeks reduced macrophage infiltration in the adipose tissues and expression of pro-inflammatory adipokines. Concomitantly, anti-inflammatory adipokines expression was increased [91]. Likewise, δ-tocotrienol reduces fat cell hypertrophy and decreases inflammation in both liver and adipose tissue in mice with obesity [91]. δ-Tocotrienol effectively suppresses the production of nitric oxide induction in macrophages from four different strains of mouse models [92].

γ-Tocotrienol possesses antioxidant and anti-inflammatory functions in vivo as well. Research has shown the ability of γ-tocotrienol to increase the expression of endogenous antioxidant enzymes, such as Gpx and superoxide dismutase (SOD) [57,93]. In young C57BL/6J mice with high-fat diet-induced obesity and insulin resistance, 0.05% γ-tocotrienol supplementation for 4 weeks reduced plasma pro-inflammatory cytokines and the recruitment of adipose tissue macrophages (ATMs). The macrophage-specific markers, MCP-1, the levels of pro-inflammatory IL-6 and IL-1b in epididymal fat, and plasma levels of inflammatory cytokines, including IL-6, IL-9, IL-10, IL-12, and TNF-α, were also suppressed by γ-tocotrienol [82]. A recent study indicates that γ-tocotrienol supplementation suppresses high-fat, high-cholesterol diet-induced hepatic inflammation in C57BL/6 male mice by downregulating the expression of MCP-1, CD11c, TNF-α, NLRP3, and IL-1β [94].

The anti-inflammatory activity of tocotrienols may be directly applicable in COPD. In a rat model of COPD induced by cigarette smoking, γ-tocotrienol reduced inflammation in the lung tissue and prevented the decline of lung function [47]. Oral gavage of γ-tocotrienol reduced neutrophil counts in a dose-dependent manner in rats exposed to cigarette smoke [47]. In addition, γ-tocotrienol inhibited nuclear translocation of STAT3 and NFκB and increased nuclear erythroid 2-related factor 2 (Nrf2) translocation [47]. Oral γ-tocotrienol supplementation protected mice against airway remodeling and emphysema and improved overall lung function to higher extents than prednisolone, the standard anti-inflammation treatment option [47]. Similarly, the γ-tocotrienol modulates allergic response through the neutralization of free radicals, the inhibition of NFκB activation, and the promotion of Nrf2 activation in an asthma mouse model [93]. Furthermore, tocotrienol prevents inflammation and edema in an asthmatic rat model [95]. Given the ability of γ-tocotrienol to target the components of COPD, such as cytokine production and inflammation, γ-tocotrienol is a possible adjuvant agent in preventing the progression of COPD and decreasing the associated risk of lung cancer development.

## 8. Antioxidant and Anti-Inflammatory Effects of Tocotrienol in COPD: Human Studies

The majority of previous research on vitamin E and lung function has been focused on the effects of tocopherols. A large randomized trial with 38,597 women for ten years found 600 IU tocopherol supplement led to a 10% reduction in the risk of chronic lung disease [96]. Epidemiological studies show that increased intake of vitamin E is associated with decreased development of COPD in both smokers and non-smokers [74,97]. Vitamin E intake is also positively correlated with higher FEV1 and negatively correlated with COPD mortality rate [97,98,99]. A clinical study indicated that increased intake of vitamin E-enriched foods could decrease the serum level of carbonyl, a marker of inflammation [100]. An independent human study demonstrated that an increased intake of vitamin E, abundant in plant foods such as fruits, vegetables, nuts, seeds, and oils, provides antioxidant and anti-inflammatory effects [101] and decreases the COPD mortality rate by 23% [74]. Previous studies also showed that diet with vitamin E could mitigate cough in patients with COPD [102] and long-term vitamin E supplementation reduced markers of oxidative stress in the urine from male cigarette smokers [103]. Additionally, a clinical study reported that vitamin E intake protected patients from wheezing, a symptom of COPD, in the Netherland [104]. Similarly, studies indicated that a higher intake of α-tocopherol conferred a modest protective effect on adult-onset asthma and a beneficial effect on lung function in Finland and Italy [105]. Another study found that lung function, and single-breath nitrogen washout, were not significantly different between vitamin E and placebo groups [106]. A large cohort study over 10 years found subjects in the highest quintile of tocopherol intake had a relative risk of 0.53 for asthma compared with subjects in the lowest quintile [76].

Other clinical trials have shown conflicting results. One clinical trial showed 800 IU of tocopherol supplement per day for eight weeks did not improve clinical parameters in patients with COPD [107]. A study with 2633 adults demonstrated an intake of 2.2 mg/day vitamin E is positively associated with a higher FEV1 and FVC ratio [108]. However, the same study indicates that vitamin C and vitamin E intakes were significantly correlated, and after allowing for the effects of vitamin C there was no additional independent effect of vitamin E on either FEV1 or FVC. In addition, an independent study showed that eight-week vitamin E supplementation did not improve the lung function of patients with COPD [109]. However, in this study, tocopherol supplementation lowers the level of lipid peroxide and elevated the concentrations of antioxidants in the plasma.

While there is no clinical trial to study the preventive effects of tocotrienol on the development of COPD, there are a limited number of human studies investigating the anti-inflammatory activity of tocotrienols. A pilot study examined the effects of δ-tocotrienol supplementation in a cohort with non-alcoholic liver disease (NAFLD) [110]. δ-Tocotrienol significantly decreased biomarkers of inflammation and oxidative stress, such as high sensitivity-CRP (hs-CRP) and malondialdehyde (MDA). In a separate study, the mRNA levels for the pro-inflammatory TNF-α and VCAM-1 in plasma from patients fed 500 mg δ-tocotrienol per day for 6 weeks were decreased by 47% and 22%, respectively [111]. A low dose of TRF at 60 mg per day reduced oxidative stress in non-familial hypercholesterolemia patients as shown in the reduction of MDA, F2-isoprostanes, and oxidized LDL (ox-LDL) [112]. In healthy adults aged 50–55, 150 mg TRF per day reduced MDA and DNA damage and offered stronger protection against oxidative damage than α-tocopherol [113]. RNA sequencing data indicated that oral supplement of δ-tocotrienol in patients with chronic hepatitis C reduces the expression of multiple genes such as the EIF2, mTOR, and TNF-α pathways [111]. The inclusion of a larger number of patients and supplementation with different formats of vitamin E (tocotrienol vs. tocopherols) for longer periods may shed more light on how the antioxidant and anti-inflammatory activities of tocotrienols may offer protestation against COPD.

## 9. Availability, Metabolism, and Safety of Tocotrienols

Tocopherols are found in the majority of plant-based foods. In comparison, tocotrienols are generally located in seeds, such as annatto seeds, and fruits, such as palm fruits, with a relatively higher variation and lower level of abundance [114]. All forms of vitamin E are absorbed in the small intestines. Following absorption, vitamin E molecules are packaged into chylomicrons and delivered to the liver or peripheral tissues. Chylomicrons are acted upon by lipoprotein lipase to allow for tissue uptake of vitamin E. Following hepatic uptake, vitamin E in chylomicron remnants is incorporated into very-low-density lipoprotein (VLDL) by the α-tocopherol transfer protein (α-TTP) and transported in circulation to peripheral tissues and vital organs [115,116]. In comparison to other vitamin E isoforms, α-tocopherol has the greatest binding affinity for α-TTP, resulting in the highest amounts of α-tocopherol in circulation. Unbound forms of vitamin E such as tocotrienols are susceptible to catabolism through ω-hydroxylation mediated by hepatic cytochrome P450 ω-hydroxylase [57,71]. Tocotrienols are further metabolized through β-oxidation to carboxychromanols, hydroxycarbooxychromanol, and carboxyethylhydroxychroman derivatives [57,71].

Plasma tocotrienol levels are reported to peak at 3746 ng/mL, or 9.8 μM at 4–9 h after ingestion of 1600 mg δ-tocotrienol [117]. The intake of 1600 mg twice daily did not lead to any toxicities [117,118]. A separate feeding study in healthy human subjects showed that 500 mg/d of δ-tocotrienol led to a similar peak level of 3278 ng/mL, or 8.6 μM, at 6 h after ingestion [118]. Other studies with lower doses of tocotrienols reported lower peak plasma levels [119,120,121]. The half-life for tocotrienols range between approximately 2–7 h [117,118], and supplementation twice a day is recommended to maintain an optimal plasma level. The dose of tocotrienol used in this study was much higher than was used in previous studies showing the anti-inflammatory and antioxidant effect of tocotrienol [118,122], suggesting that tocotrienols could be used to prevent the progression of COPD. Data from phase II trials in recurrent ovarian cancer patients also show tocotrienol improves survival with low toxic effects (300 mg × 3/day) [123].

The question remains if α-TTP is the only mechanism through which tocotrienols reach tissues and vital organs. In order to understand the significance of α-TTP in α-tocotrienol delivery, levels of α-tocotrienol in the tissue were measured in α-TTP-deficient mice supplemented with α-tocotrienol [124]. Interestingly, this study showed that α-tocotrienol was transported to several vital organs in α-TTP-deficient mice. Besides, α-tocotrienol restored fertility in α-TTP-deficient mice [124], suggesting that high doses of tocotrienol (5 mg/kg) by gavage may overcome the necessity for α-TTP. Given the low solubility of tocotrienol in plasma, these results suggest that an independent mechanism apart from α-TTP transport may exist for the delivery of tocotrienol to the peripheral tissues.

## 10. Limitation

While the cell culture, animal studies, and preliminary human trials have shown the anti-inflammatory and antioxidant activities of tocotrienols, more work is needed, particularly on their bioavailability, for tocotrienol supplements to be effective in COPD prevention. Due to the unique structure of their phytyl tail compared to tocopherol, tocotrienols have low bioavailability. In addition, the enrichments of tocotrienols differ across different types of natural products. For example, barley has a higher percentage of α-tocotrienol, while palm oil and annatto fruit have higher γ- and δ-tocotrienol respectively [125,126]. A pharmacokinetics study found α-, γ-, and δ-tocotrienol have different bioavailability (28%, 9%, 9%) in rats. The biological half-lives of α-, γ-, and δ-tocotrienol are significantly shorter than that of α-tocopherol, which affects their levels in circulation and tissue absorption rates [127]. Multiple studies indicate tocotrienols accumulate in the adipose tissue, lung, skin, spleen, heart, kidney, muscle, and bone marrow with the highest amounts in adipose tissue [127,128,129,130]. Nevertheless, novel formulations including self-emulsifying drug-delivery systems [131] and nanotechnology [132] may enhance the bioavailability and targeted tissue delivery of tocotrienols. Tocotrienol derivatives with higher potencies could be examined [133]. The combination of tocotrienols with other compounds with complementary anti-inflammatory and antioxidant activities may also augment the efficacy of tocotrienols. Future human trials are needed to demonstrate the effectiveness of tocotrienols and elucidate their mechanisms of action.

## 11. Conclusions and Future Directions

COPD is a highly preventable disease with exceptionally high mortality rates around the world. In addition, COPD serves as a significant risk factor for lung cancer development, especially in individuals with a history of cigarette smoking, which is also the primary risk factor for COPD development. The pathology of COPD involves an inflammatory response locally within the lung tissue, contributing to the progression of the disease and the development of comorbidities. Although smoking cessation and the primary prevention of cigarette smoking are critical for the long-term reduction of COPD risk, most patients with COPD have smoking history [134]. There is an urgent need for effective chemoprevention and therapeutic strategies other than smoking cessation. Current treatment options for COPD remain ineffective in reducing the severity of the disease and have undesirable side effects, leaving clinicians and researchers yearning for alternative preventative and treatment approaches. Cigarette smoking contains many free radicals and carcinogenic compounds. These toxins initiate inflammatory effects by activating the NFκB pathway. The activated NFκB pathway leads to a cascade of inflammation in the airway epithelial cells and the recruitment of macrophages and neutrophils. Therefore, the persistence of inflammation mediated by the NFκB pathway not only inaugurates the phenotypes of COPD but may also exacerbate the status of the disease.

Tocotrienols, a group of four vitamin E molecules with antioxidant and anti-inflammatory capabilities, reduce inflammation within and improve the function of the lung tissue of animals with cigarette-smoking-induced COPD. Tocotrienols block NFκB activation by preventing the phosphorylation of IκB mediated by the IKK complex, decreasing IκB degradation, and blocking the translocation of NFκB into the nucleus, thereby suppressing the activation of the inflammatory pathway [77]. Further in vitro and in vivo studies are needed to investigate the role of anti-inflammatory pathways, the secretion of cytokines, and the progression of COPD. Given the importance of inflammation and oxidant stress in patients with COPD, tocotrienols with strong antioxidant, anti-inflammatory activities, and safety profiles could be an alternative prevention strategy for the development of COPD.

## Figures and Tables

**Table 1:** Cell culture, animal, and human studies showing the antioxidant and anti-inflammatory activities of tocotrienols.

Model	Vitamin E Types	Mechanism	Reference
Cell Culture			
rat liver microsomes	α-tocotrienol	α-Tocotrienol has 40–60 times higher antioxidant activity than α-tocopherol.	[66]
human lung carcinoma A549 cell	α- and γ-tocotrienol	Both isoforms reduce ROS formation, lipid peroxidation, cytokine production, and apoptosis; γ-tocotrienol ameliorates the LPS-induced reduction in cell viability.	[72]
human myeloid KBM-5 cells, lung adenocarcinoma cells H1299, breast cancer MCF7, multiple myeloma U266, squamous cell carcinoma SCC4	γ-tocotrienol	🠗 NFκB activation by different stimulants (LPS, EGF, TNF-α)	[73]
Human lung cancer cell lines A549 and	δ -tocotrienol	🠗 cancer cells proliferation, migration and invasion	[74,75,76]
LPS-induced inflammation in human umbilical vein endothelial cells (HUVECs)	δ- and γ-tocotrienol	🠗 IL-6, ICAM-1, VCAM-1 and NFκB; 🠗 e-selectin and eNOs;	[77]
RAW 264.7 macrophages and peritoneal macrophages isolated from LPS-treated BALB/c mice	α-, δ-, and γ-tocotrienol	🠗 TNF-α dose-dependently; 🠗 gene expression of TNF-α, IL-1β, IL-6, iNOS; δ -tocotrienol is most effective	[78]
iJ774 macrophages	δ-tocotrienol	🠗 ROS, IL-1β, NLRP3 inflammasome	[79]
murine RAW 264.7 macrophages and primary bone marrow-derived macrophages	γ-tocotrienol	🠕 inhibitors of NFκB (A20, Cezanne) and de novo synthesis of sphingolipid	[80]
bone marrow derived macrophage	γ-tocotrienol	🠗 glucose intake 🠗 LPS-induced NFκB activation	[81]
bone marrow derived macrophage	γ-tocotrienol	🠗 CD11b, TNF-α, IL-6, and IL-1β 🠗 LPS-induced M1 macrophage polarization and NFκB activation	[82]
LPS-induced inflammation in murine RAW 264.7 macrophages	γ-tocotrienol	🠗 IL-6 and G-CSF production through the inhibition of NFκB and C/EBPβ pathways.	[83]
murine RAW 264.7 macrophages	tocotrienol rich fraction; α-, δ-, and γ-tocotrienol	🠗 production of inflammatory products (TNF-α, IL-6, NO, COX-2)	[84]
murine RAW 264.7 macrophages.	δ-tocotrienol	🠗 NFκB activation 🠗 phosphorylation of TAK1 🠕 up-regulation of A20 and CYLD	[85]
osteoblastic cells	δ-tocotrienol	🠗 NFκB downstream target	[86]
murine 3T3-L1 adipocytes	δ-tocotrienol	🠗 secretion of adipokines IL-6 🠗 JNK inflammation pathway 🠗 NFκB activation	[87]
murine 3T3-L1 adipocytes	γ-tocotrienol	🠗 TNF-α in adipocytes; 🠗 secretion of adipokines IL-6, MCP-1 🠗 NFκB activation	[88]
human adipose-derived stem cells	muscadine grape seed oil containing α- and γ-tocotrienol	🠗 LPS-induced IL-6, IL-8, and MCP-1	[89]
**Animal Study**			
cigarette-smoke induced model of COPD in a rat model	γ-tocotrienol	🠗 reduces cigarette smoke-induced BAL fluid neutrophil counts and inflammatory cytokine concentrations dose-dependently	[47]
metabolic syndrome and bone loss in rats	palm tocotrienol	🠕 skeletal-promoting benefit by modulating the levels of osteocytes- derived bone-related peptides	[65]
BALB/c mice	α-, δ-, and γ-tocotrienol	🠗 TNF-α, IL-1β, IL-6 and iNOS	[78]
obesity induced-inflammation and insulin resistance in C57BL/6J mice	γ-tocotrienol	🠗 body weight gain, improved insulin signaling 🠗 MCP-1 and macrophage recruitment into adipose tissue	[82]
obesity in mice	δ-tocotrienol	🠗 fat cell hypertrophy and inflammation in both liver and adipose tissue	[90,91]
C57BL/6, BALB/c, LMP7/MECL-1^-/-^, and PPARα^-/-^ mice	δ-tocotrienol	🠗 TNF-α, iNOS induction, and NO production.	[92]
house dust-mite- mediated asthma model in BALB/c mice	γ-tocotrienol	🠗 productions of free radicals, cytokines, chemokines, ROS, oxidative damage biomarkers, NFκB 🠕 nuclear Nrf2, endogenous antioxidant activity	[93]
C57BL/6 male mice with high fat diet inducing nonalcoholic fatty liver disease (NAFLD)	γ-tocotrienol	🠗 productions reduce the diet-induced hepatic ER stress and fibrosis	[94]
C57BL/6 male mice ovalbumin (OVA)-challenged asthmatic brown Norway rats	γ-tocotrienol palm oil tocotrienol-rich fraction (TRF)	🠗 hepatic inflammation and the expression of MCP-1, CDE11c, TNF-α, NLRP3, and IL-1β 🠗 reduces edema and inflammatory cell infiltration in the bronchial wall	[94,95]
**Human Chronic and Intervention Studies**			
2917 men aged 50–69 y	Dietary Vitamin E	Vitamin E intake prevent the development of COPD	[74]
Total 38,597 women without chronic lung disease in the Women’s Health Study (WHS)	randomised double-blind placebo-controlled factorial trial of vitamin E (600 IU every other day)	600 IU vitamin E led to a 10% reduction in the risk of chronic lung disease in women	[96]
115 COPD patients and 115 controls	Spirometry and food questionnair were used	fruit and vegetable consumption is inversely associated with chronic obstructive pulmonary disease	[97]
MORGEN study 13 651 men and women aged 20–59 years for 20 years	fruit, vegetable, fish, alcohol, and whole grain consumption	independent beneficial effects of fruits, whole grains on COPD	[98]
40 male smokers with clinical diagnosis of COPD (Group-I (GI)) and 36 healthy smokers without COPD	65 food items from five main food groups (grain, meat and alternatives, dairy products, vegetables-fruits, and fat) and 25 dietary habits.	Dietary intake of black tea and vegetables-fruits consumptions may be protecting male smokers from developing COPD	[99]
A total of 267 patients with COPD	Dietary data of the last 2 years was assessed using a validated food questionnaire	dietary vitamin E intake prevents systemic oxidative stress in COPD patients, particularly in those that continue smoking.	[100]
196 Scottish Men in smoker and non-smokers	Food Frequency Questionnaire and validated by serum samples	Both dietary and serum values of vitamin E were lower in smokers than non-smokers	[101]
84 patients with COPD and 80 controls	Dietary Approaches to Stop Hypertension (DASH) diet	DASH dietary pattern among patients with COPD was significantly lower compared to the control group. Cough was significantly decreased by increments in adherence to a DASH dietary	[102]
Total 35,533 subjects	randomized vitamin E supplementation	Vitamin E supplementation decreases urine 8-iso-PGF2α among male cigarette smokers	[103]
Dutch population on 6555 adults during 1994 and 1995	Asemi-quantitative food frequency questionnaire and respiratory symptoms were assessed by a self-administered questionnaire.	Vitamin E intake showed no association with most symptoms and lung function, but had a positive association with productive cough.	[104]
Subjects data were collected in the 1960s in Finland (1248), Italy (1386), and the Netherlands (691)	The cross-check dietary history method was used and lung function was measured by the Spirometry	Associations of vitamin E with pulmonary function were not consistent across countries	[105]
young healthy adult volunteers exposed for 2 h periods to 0.5 ppm O_3_, with secondary stresses of heat and intermittent light exercise.	Subjects received 800 or 1600 IU vitamin E per day for 9 or more wk	There is not significantly difference between vitamin E and placebo groups	[106]
82 patients with COPD and 22 healthy non-smoking controls	Lung function was measured by spirometry.	systemic oxidant-antioxidant imbalance in the patients with COPD.	[107]
2633 subjects 18 to 70 yr	Vitamin E by semiquantitative food frequency questionnair	higher intake of vitamin E was associated with better lung function	[108]
Thirty patients with COPD with 12 weeks of supplementation with 400 IU of vitamin E daily	spirometry, plasma malondialdehyde (MDA), SOD levels were measured	Vitamin E supplementation does not have any significant effect on lung function but it lowers the levels of MDA	[109]
71 patients with non-alcoholic fatty liver disease (NAFLD)	oral supplementation of δ-tocotrienol	🠗 hs-CRP, MDA, and FLI score in comparison to the placebo group	[110]
14 patients with hepatitis C	δ-tocotrienol (500 mg/d) for 6 weeks	🠗 δ-tocotrienol inhibits multiple-signaling pathways such as TNF-α, LMP2, 7 and 10, IFN-γ, ICAM1, VCAM1 without any side-	[111]
non-familial hypercholesterolmia patients	60 mg/d TRF	🠗 MDA, F2-isoprostanes, ox-LDL	[112]
71 subjects both male and female aged between 50 and 55 years	plasma MDA, protein carbonyl, total DNA damage, vitamin D concentration and vitamin E isomers were measured	🠗 Tocotrienol supplementation effects were different from α-tocopherol in reducing oxidative stress markers	[113]

Abbreviations: FLI: fatty liver index; G-CSF: granulocyte-colony-stimulating factor; GM-CSF: Granulocyte-macrophage colony-stimulating factor; hs-CRP: high-sensitivity C-reaction protein; IL-1β: interleukin-1β; IL-6: interleukin-6; iNOS: inducible nitric oxide synthase; LPS: lipopolysaccharide; MDA: malondialdehyde; MCP-1: monocyte chemoattractant protein-1; NO: nitric oxide; NFκB: nuclear factor kappa B; Nrf2: nuclear factor erythroid 2; ROS: reactive oxygen species; STAT3: signal transducer and activator of transcription 3; TNF-α: tumor necrosis factor-α; VCAM-1: vascular adhesion protein 1.

## Abbreviations

G-CSF	granulocyte-colony-stimulating factor;
hs-CRP	high-sensitivity C-reaction protein;
IL-1β	interleukin-1β;
IL-6	interleukin-6;
iNOS	inducible nitric oxide synthase;
LPS	lipopolysaccharide;
MDA	malondialdehyde;
MCP-1	monocyte chemoattractant protein-1;
NO	nitric oxide;
NF-kB	nuclear factor kappa B;
Nrf2	nuclear factor erythroid 2;
ROS	reactive oxygen species;
STAT3	signal transducer and activator of transcription 3;
TNF-α	tumor necrosis factor-α;
VCAM-1	vascular adhesion protein.
